# Exploring the Therapeutic Potential of Berberine and Tocopherol in Managing Diabetic Neuropathy: A Comprehensive Approach towards Alleviating Chronic Neuropathic Pain

**DOI:** 10.3390/biomedicines11061726

**Published:** 2023-06-15

**Authors:** Faisal K. Alkholifi, Alhussain H. Aodah, Ahmed I. Foudah, Aftab Alam

**Affiliations:** 1 Department of Pharmacology & Toxicology, College of Pharmacy, Prince Sattam bin Abdulaziz University, Al-Kharj 11942, Saudi Arabia; 2 Department of Pharmaceutics, College of Pharmacy, Prince Sattam bin Abdulaziz University, Al-Kharj 11942, Saudi Arabia; a.aodah@psau.edu.sa; 3 Department of Pharmacognosy, College of Pharmacy, Prince Sattam bin Abdulaziz University, Al-Kharj 11942, Saudi Arabia; a.foudah@psau.edu.sa (A.I.F.); a.alam@psau.edu.sa (A.A.)

**Keywords:** diabetes, neuropathic pain, Berberine, Tocopherol, diagnostic evaluation, pain management

## Abstract

Diabetic neuropathy (DN) causes sensory dysfunction, such as numbness, tingling, or burning sensations. Traditional medication may not ease pain and discomfort, but natural remedies such as Berberine (BR) and vitamin E or Tocopherol (TOC) have therapeutic potential to reduce inflammation while improving nerve function. Novel substances offer a more potent alternative method for managing severe chronic neuropathic pain that does not react to standard drug therapy by targeting various pathways that regulate it. Rats with diabetic control received oral doses of BR + TOC that showed significant changes in serum insulin levels compared to DN controls after 90 days, suggesting a decrease in sensitivity to painful stimuli partly by modulating the oxidative stress of the inflammatory pathway such as TNF-α suppression or stimulation of TNF-α depending on the amount of dose consumed by them. NF-kB also played its role here. Administering doses of BR and TOC reduced heightened levels of NF-kB and AGEs, effectively counteracting inflammation-targeted key factors in diabetes, promising possibilities for the benefits of these molecules revealed through in vivo investigation. In summary, treating neuropathy pain with a more comprehensive and organic approach can involve harnessing the powerful capabilities of BR and TOC. These compounds have been found to not only considerably decrease inflammation but also provide effective nerve protection while enhancing overall nerve function. With their multifunctional impacts on various neuropathic pain pathways in the body, these naturally occurring substances offer an exciting possibility for those who encounter high levels of neuropathic distress that do not respond well to conventional medication-centred therapies.

## 1. Introduction

Diabetic neuropathy (DN) is an important risk of hyperglycaemia that damages nerves and causes severe disease, affecting patients’ quality of life with symptoms such as tingling of the limb, numbness, muscle weakness, digestive issues (e.g., diarrhoea or constipation), and UTI. Regular glucose monitoring and check-ups can prevent DN in diabetics [1,2,3,4]. DN is a prevalent complication observed in almost 40% of individuals with poorly managed diabetes [5].

DN pathogenesis is controversial. Due to the many factors that can cause this debilitating disease, genetics, environmental conditions, lifestyle, and comorbidities interact [6,7,8,9]. The latest research has unveiled a new dimension of the gut microbiota in the pathophysiology of DN. Modifications within the gut microbiota have shown their significant role in host metabolism, immune system function, and systemic inflammation, which are directly related to neuropathy. A connection appears to be established between changes detected in intestinal microbial populations and patients with diabetes or neuropathic symptoms, as such individuals exhibit dysbiosis [10]. DN increases pathogens and intestinal microbiota diversity [11].

Long-term high blood glucose levels cause the complex metabolic pathway that leads to DN [12,13]. Hyperglycaemia triggers metabolic reactions through pathways such as ALR-polyol, hexosamine, and AGE. A proper understanding of these mechanisms is crucial to managing diabetes mellitus [14,15]. DN involves oxidative-nitrosative stress that leads to damage to lipids, DNA, and proteins. Diabetes lowers antioxidants and alters gene expression patterns, causing inflammation and nerve dysfunction. Thorough research helps identify therapeutic targets to prevent negative effects from DN [16,17,18,19,20,21,22]. Antioxidants defend cells from reactive oxygen and nitrogen species (ROS/RNS). They are important for health and protect against harm by neutralising toxic chemicals with antioxidant enzymes such as glutathione reductase (GSH) and superoxide dismutase (SOD), both endogenous and exogenous [23,24,25,26,27].

Antioxidants and bioactive food components have health benefits. A balanced diet meets the nutrient needs of good health [28,29]. Antioxidants and bioactive foods offer health benefits, highlighting the importance of a balanced diet for overall well-being [30,31]. Eating antioxidant-rich natural foods reduces the risk of premature death and protects against chronic diseases such as cancer, rheumatoid arthritis, cardiovascular disease, diabetes, and neurological disorders. Antioxidants play an important role in maintaining our health by enhancing immune system function. Eating fruits and vegetables with high antioxidants can help combat severe diseases while promoting better overall health [32,33,34,35,36]. Inflammation causes diabetes complications through cytokines and chemokines [37]. Recent research found that cytokine-reducing drugs and antioxidants have potential benefits in the treatment of diabetes. Functional foods and supplements are also being explored to regulate inflammation and oxidative damage. Investigating these mechanisms can improve diabetes management and develop new therapies at the molecular level [38,39,40,41].

Berberine, with immense health benefits and anti-inflammatory properties, makes it a natural therapy choice without adverse effects. This compound is versatile due to its antioxidant, anti-stress, and antitumor activities and is popular among health-conscious individuals who value the healing potential of nature [42]. More recently, it was discovered that Berberine also has an impact comparable to that of an antidiabetic and has been used in clinical investigations to treat type 2 diabetes [43]. Berberine has been shown to alleviate some of the symptoms of diabetic nephropathy by reducing inflammatory responses, avoiding podocyte death, and modifying the activity of the nuclear factor kappa B complex [44]. Recent results from a variety of clinical tests have shown that Berberine is an effective drug for reducing diabetic neuropathy [42]. Furthermore, Berberine was found to be helpful in reducing visceral pain and improving intestinal motility in mice models of irritable bowel syndrome [45].

Vitamin E, also known as Tocopherol (TOC), is a powerful antioxidant that possesses the anti-inflammatory qualities necessary to achieve optimal bodily functions. This vital nutrient can even protect the nervous system by serving as a neuroprotective agent. Failing to incorporate TOC into one’s diet can cause inadequate neural development that could adversely affect overall health and wellness. Therefore, it is imperative not to overlook this critical component of nutrition [46]. Undoubtedly, it cannot be overstated that TOC is of utmost importance, as it serves a vital function in promoting optimal embryo development, stimulating brain cell growth, amplifying cognitive abilities, and protecting against detrimental free radical damage [47,48]. TOC has an exceptional ability to function as a lipophilic antioxidant in LOX, which enables it to protect against cell death caused by ROS and oxytosis. Therefore, adding this nutrient to one’s daily diet or supplement routine can serve as a powerful defence mechanism in protecting one’s valuable cells from potential harm. Through the inclusion of iron in nonhaeme enzymes, particularly lipoxygenases (LOXs), intricate reactions can be catalysed that involve the deoxygenation of polyunsaturated fatty acids (PUFAs). These critical enzymes are vital players in various biological processes, including inflammation and cell signalling. Evidence suggests that TOC possesses antioxidant properties and is capable of stabilising and preserving cellular membranes, making it an advantageous aid for individuals with DN who require ongoing care. This demonstrates how enzymatic pathways intersect with essential nutrients necessary for optimal physiological performance [49].

This study investigates a potentially promising treatment solution for diabetic neuropathy by investigating the effects of Berberine and Tocopherol on the alleviation of chronic pain. This research is noteworthy because it explores how these two compounds, known for their antidiabetic, neuroprotective, antioxidant, and anti-inflammatory properties, complement each other to provide a synergistic effect in addressing this condition from several angles, such as nerve damage, oxidative stress, and inflammation, making it an innovative approach for comprehensive management of diabetic neuropathy. The study explores the potential therapeutic benefits of using Berberine and Tocopherol for the treatment of diabetic neuropathy. This research provides information on identifying effective strategies to alleviate pain associated with the disease, taking a natural approach. The investigation boldly proposes harnessing the power of naturally occurring compounds in medicinal plants, such as Berberine and Tocopherol, that are not only affordable but also well-tolerated treatments. This broader understanding promises new avenues of therapy that can enhance our fight against chronic diseases such as diabetes.

This aligns with the interest in alternative therapies that offer a safer approach to the treatment of diabetic neuropathy. Our investigation focuses on measuring fasting blood glucose, body weight, serum insulin, as well as NF-kB levels to gather comprehensive data. Overall, this study boldly explores natural compounds and their synergistic effects as potential therapeutic agents. It could advance the understanding and treatment of chronic neuropathic pain caused by diabetic neuropathy.

## 2. Materials and Methods

### 2.1. Chemicals

Chemicals such as sulphanilamide, phosphoric acid, N-(1-Naphthyl)-Ethylenediamine, β-Nicotinamide adenine dinucleotide phosphate hydrate (NADH) used were analytical grade and received as a gift from the pharmaceutics department, College of Pharmacy, King Saud University (KSU), Riyadh, Saudi Arabia. Other compounds such as Berberine chloride, Tocopherol, bovine serum albumin (BSA), streptozotocin, trichloroacetic acid (TCA), 5,5′-dithiobis(2-nitrobenzoic acid) or DTNB, nitrogen blue tetrazolium chloride (NBT), and thiobarbituric acid (TBA) were purchased from Sigma-Aldrich (St. Louis, MO, USA). TBARS kits were obtained from Abcam (Cambridge, UK), and tumour necrosis factor (TNF-) assay kits of tumour-necrosis factor (TNF-α) assay were obtained from R&D Systems, Inc. (McKinley Place NE, Minneapolis, MN, USA), while NF-kB was obtained from Santa Cruz Biotechnology, Incorporated (Dallas, TX, USA). All the other chemicals were of analytical grade and used from the College of Pharmacy, Prince Sattam bin Abdulaziz University (PSAU), Al-Kharj, Saudi Arabia.

### 2.2. Animals

The experimental protocol received approval from the Institutional Animal Ethics Committee. Rats were fed standard rodent food and had access to tap water ad libitum. The animal care and treatment protocols used in this study adhere to the most stringent standards set by the Animal Care Guidelines of the Standing Committee on Bioethics of Prince Sattam Bin Abdulaziz University (PSAU). Our unwavering dedication to ethical responsibility is attested by approval number SCBR-024-2022, which confirms that all research activities involving animals are carried out with the utmost consideration and care. The animals were kept according to the recommendations made by the CPCSEA. Wistar rats (male), weight 250–300 g, were kept in conventional environmental circumstances. These settings included a light and dark cycle (12 h) and a temperature of 23 ± 2 °C.

### 2.3. Induction of Diabetic Neuropathy

#### 2.3.1. Experimental Protocol

**Figure 1:**
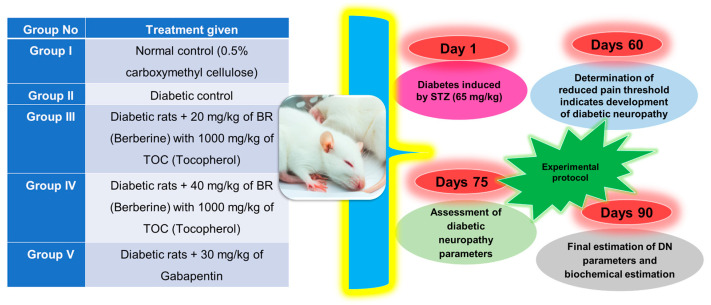
Experimental protocol of animals’ study.

The grouping of animals and experimental protocol for diabetic nephropathy is given in (Figure 1) [50].

#### 2.3.2. Estimation of Parameters

At the beginning of the experiment, we pay attention to various factors, such as individual animal body weight and blood glucose levels, along with serum insulin estimates. We began by establishing baseline serum insulin levels through rigorous measurements. The animals were housed within comparable ranges with respect to their body mass to ensure precision in the testing conditions. Randomisations were performed prior to implementing the treatment protocol to eliminate biases that could affect the results. Throughout this study, regular checks on each group’s animal’s body weight were consistently performed for accuracy purposes. Commercial enzymatic kits were used throughout the study aimed at determining fasting blood sugar levels [51,52,53].

### 2.4. Observation of Behaviour

#### 2.4.1. Evaluation of the Heat Sensitivity of Pain

To carry out the experiment successfully, we subjected a rat’s tail to heat by immersing it in water that was kept at precisely 52.5 °C ± 0.5 °C. We continued heating until the rat showed signs of discomfort, such as pulling or retracting its tail, which had a cut-off time limit of no more than 12 s for safety purposes. It is vital to note that observation of reduced reaction times during withdrawal indicates hyperalgesia and adds credibility to our findings regarding pain perception in rats under these conditions [54].

#### 2.4.2. Hot-Plate Method

Using the hot-plate, one experiences a hyperalgesic reaction, which is hypothesised to be caused by central and peripheral mechanisms. The temperature of Eddy’s hot-plate was maintained at 55 °C, and each animal was assigned its own spot on the plate. The length of time that passed before the animals began to lick their paws or jump to escape the heat served as the basis for calculating the pain threshold (cut-off time was 10 s) [55].

#### 2.4.3. Evaluation of the Level of Mechanical Hyperalgesia

During the Randall–Selitto test, the analgesic metre was used to record the amount of pressure placed on the lower extremities. A linear rate of 10 g/s was used to bring the pressure to the centre of the hind paw. The pressure was limited to 250 grammes to prevent any injury to the surrounding tissue from developing. A total of five tests were performed on each animal at intervals of at least 15 min, and the mean value of these tests was then calculated afterwards [56].

#### 2.4.4. Evaluation of the Sensation of Allodynia

The withdrawal threshold of the hind paw of the animal was examined in response to stimulation with von Frey filaments to determine how sensitive the animal is to tactile stimulation. This was performed by stimulating the paw with the filaments. After being compartmentalised into their own individual boxes and laid on a stainless steel mesh floor, rats were given at least 20 min to adapt themselves to their brand-new surroundings. When applied perpendicularly to the plantar surface of a hind paw with enough power to force the filament to bend for six seconds, a series of calibrated von Frey filaments varying in weight from 0.4 to 64 gm were used. The force was administered in this manner for the duration of the study. When the animal removed its paw or flinched in response to stimuli, this was deemed a favourable response [57,58,59].

#### 2.4.5. Homogenising Tissue

Various tissue groups were collected and thoroughly washed with saline solution to obtain accurate results. These tissues were then homogenised in a buffer composed of 0.1 M Tris-HCl at pH 7.4 at 4 °C to prevent any degradation or alteration in the composition of the samples. Subsequently, centrifugation was performed for ten minutes under controlled conditions; that is, set at 300 rpm and also maintained the same low-temperature setting as before. Next, we prepared aliquots for subsequent analyses, using the BSA method to accurately determine the protein concentration contained within each sample [60].

### 2.5. Biochemical Analysis

#### 2.5.1. Estimation of Reduced Glutathione

To measure reduced glutathione levels, a highly effective technique was employed. First, the tissue homogenate supernatant was mixed with 10% weight percentage TCA and then centrifuged at a speed of 1000 rpm for precisely ten minutes at 4 °C to remove any impurities. The resulting liquid obtained after this process underwent further processing where aliquots were made and exposed to disodium hydrogen phosphate (2 mL) along with DTNB (0.001 M). Finally, an assay expressed in millimoles per milligramme of protein helped to accurately determine absorbance values, making this method efficient and reliable [61].

#### 2.5.2. Determination of Superoxide Dismutase

By introducing 50 µL of homogenate and then gradually adding distilled water, sodium pyrophosphate buffer, postmitochondrial supernatant, NBT, and NADPH (in quantities of 1.15 mL, 1.2 mL, 100 mL, 300 mL, and 200 mL, respectively), followed by the inclusion of potassium phosphate buffer measuring up to a volume of approximately 50 millilitres; we were able to stop the reaction completely using an additional dose of potassium phosphate buffer also measuring around the same volume. To determine the efficacy levels in both our control samples and those used during experimentation. Optical densities were weighed with evaluations of the enzyme–protein ratio based on milligramme measurements serving as determining factors for efficiency standards [62].

#### 2.5.3. Estimation of Lipid Peroxidation

In order to determine the final concentration in nanomoles/mg of proteins, a series of steps were taken. First, 0.2 millilitres of tissue homogenate aliquots were extracted and mixed with 0.2 millilitres of an 8.1 percent sodium dodecyl sulphate solution, as well as 1.5 millilitres each of a 30% acetic acid and a 0.8 percent thiobarbituric acid solution along with four millilitres of distilled water. Then, one more ml of distilled H_2_O was added, followed by 15 mL of the n-butanol-pyridine mixture in a ratio of 15 to one. This entire concoction was centrifuged for ten minutes at 4000 revolutions per minute before calculating its absorbance value at a wavelength level set at exactly 500-32 nanometre scale range and comparing it with previously defined standards for treated groups vs. untreated ones so that differences could be accurately quantified [63].

#### 2.5.4. Advanced Glycation End Products (AGEs) Estimation in Sciatic Nerve

The kidney was homogenised in 0.25 M sucrose and then subjected to centrifugation at 900 rounds per minute while maintaining the temperature at 5 °C. The pellets were collected, and a second round of centrifugation was performed under similar conditions. The resulting supernatant was combined with the previous one, followed by the addition of TCA to precipitate proteins which were then again subjected to centrifugation at 4 °C and 900 revolutions per minute. To remove lipids, methanol containing NaOH (1 percent) was added twice. During absorbance determination using the BSA standard curve as a reference point to measure levels of AGEs through fluorometric analysis employing emission/excitation wavelengths measured in nm (440/370 nm), the reported relative fluorescence units (RFU) results are obtained for every mg protein, which serves as persuasive evidence to support our argument [64].

#### 2.5.5. Nitrite Estimation

Using the Greiss reagent, we were able to measure the nitrate content in the supernatant collected after mitochondrial respiration. This provided us with valuable information on how much nitric oxide was produced during this process. Exactly 0.5 millilitres of Greiss reagent (consisting of a one-to-one solution of sulphanilamide and phosphoric acid along with napthylamine diamine dihydrochloric acid) was added to one-tenth of a millilitre sciatic nerve homogenate to determine nitrite levels, and its absorbance at 546 nanometres was measured. The use of sodium nitrate as a reference substance allowed standard curve construction and determination that revealed our findings in terms of milligrammes per millitre, effectively illustrating the impact our research could have on mitigating harmful effects associated with excessive NO production [65,66].

### 2.6. Examination of NF-kB and TNF-α

To detect inflammatory cytokine levels such as NF-kB and TNF-α, we acquired an ELISA kit (R&D Systems, Minneapolis, MN, USA). Every graph was formulated by consolidating all specifications provided by different manufacturers into a comprehensive report. Throughout the procedure, we followed the manufacturer’s instructions precisely to ensure accuracy. Our objective was achieved successfully with this approach [67,68].

### 2.7. Statistical Analysis

After conducting a one-way ANOVA, the statistical analysis was carried out utilising GraphPad Prism 9 programme in conjunction with Tukey’s multiple tests. The values were presented using the mean ± SEM format. Origin was used to draw the figures.

(a#): Normal control vs. diabetic control.
(b*): Diabetic control vs. 20 mg/kg and 40 mg/kg of BR (Berberine) with 1000 mg/kg of TOC (Tocopherol) and 30 mg/kg of the standard drug (Gabapentin).
(***): *p* < 0.001, (**): *p* < 0.01, and (*): *p* < 0.005.

## 3. Results

To assess diabetic neuropathy, a variety of indicators, including weight, fasting glucose, and insulin levels, were examined. Other factors, such as NF-kB and TNF-α levels, along with biochemical markers, such as AGE, SOD, and GSH, were also examined to fully understand this condition.

Diabetic neuropathy can be accurately diagnosed by observing a decrease in the pain threshold after 30 days of STZ treatment. To effectively test for DN, an experimental mixture containing Berberine and Tocopherol was orally administered to subjects. Specifically, two distinct doses of BR (20 mg/kg and 40 mg/kg) were supplemented with TOC at a dose of 1000 mg/kg. Treatment began on day 60 after the onset of DN resulting from STZ induction and lasted until day 90.

### 3.1. Effect on Body Weight

**Figure 2:**
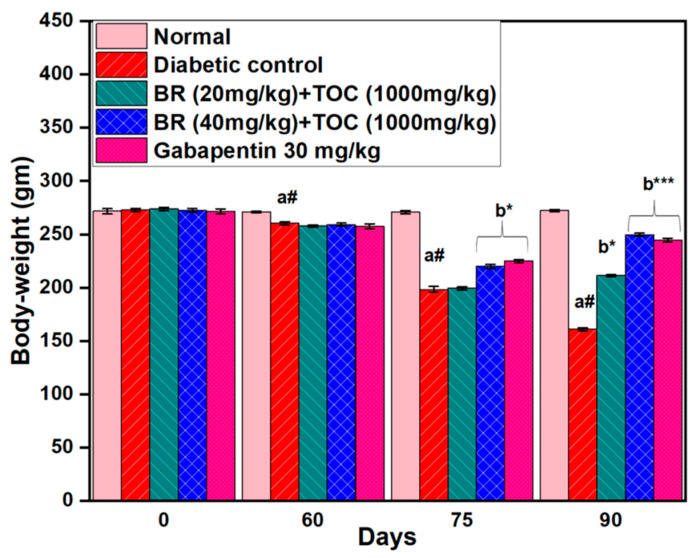
Effect of Berberine and Tocopherol on body weight. Values are represented as mean ± SEM. (a#): normal control vs. diabetic control. (b*): diabetic control vs. 20 mg/kg and 40 mg/kg of BR (Berberine) with 1000 mg/kg of TOC (Tocopherol) and 30 mg/kg of the standard drug (Gabapentin). (***): *p* < 0.001 and (*): *p* < 0.05.

Rats were weighed to initiate the investigation, and only those weighing between 250 g and 300 g were selected for further examination. Their weight was meticulously measured on four distinct occasions, at the start of the study, after a period of 60 days, after 75 days, and finally, at the end of day 90. Initially, there was no significant difference in body weight between normal control rats (272 ± 2.5 g) and diabetic control rats (273 ± 1.0 g). However, towards the end of the study period, compared with normal control rats (272 ± 1.2 g), a considerable decrease in body weight was observed among diabetic control group animals who weighed only 161 ± 1.0 g. Oral administration of BR and TOC leads to a noticeable reduction in body weight loss from day 75 to day 90. Therefore, the doses of BR and TOC (20 mg/kg and 40 mg/kg doses + 1000 mg/kg doses + 1000 mg/kg) show an improvement in the health of the animal as their final weight increases up to 211 ± 0.8 g and 250 ± 1.4 g compared with diabetic control rats 161 ± 1.0 g (*p* < 0.001) (Figure 2).

### 3.2. Effects on Fasting Blood Glucose Level

**Figure 3:**
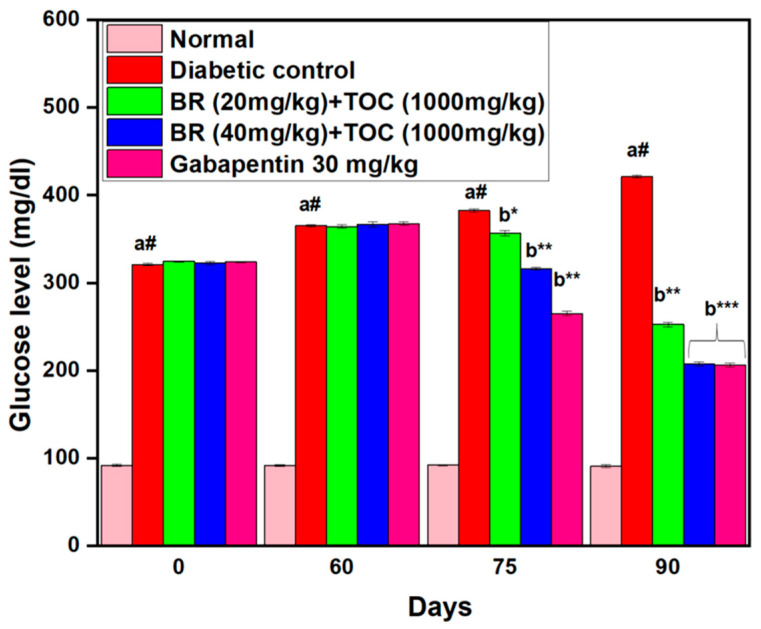
Berberine and Tocopherol on fasting blood glucose level. Values are represented as mean ± SEM. (a#): normal control vs. diabetic control. (b*): diabetic control vs. 20 mg/kg and 40 mg/kg of BR (Berberine) with 1000 mg/kg of TOC (Tocopherol) and 30 mg/kg of the standard drug (Gabapentin). (***): *p* < 0.001, (**): *p* < 0.01, and (*): *p* < 0.05.

The administration of STZ has been found to cause a considerable increase in fasting glucose levels among experimental animals. Throughout the experimentation period, which included the initial day (0th), 60th, 75th, and 90th days, glucose levels were measured at various intervals. However, with oral administration of doses of BR + TOC of 20 mg/kg and 40 mg/kg + 1000 mg/kg and Gabapentin dosage of 30 mg/kg, experimental animals exhibit significantly reduced glucose levels compared with diabetic control animals (421 ± 2.0 mg/dL) (*p* < 0.001). In particular, the extent of attenuation is found to be directly proportional to the dosage, as demonstrated by (Figure 3).

### 3.3. Effect on Serum Insulin Level

**Figure 4:**
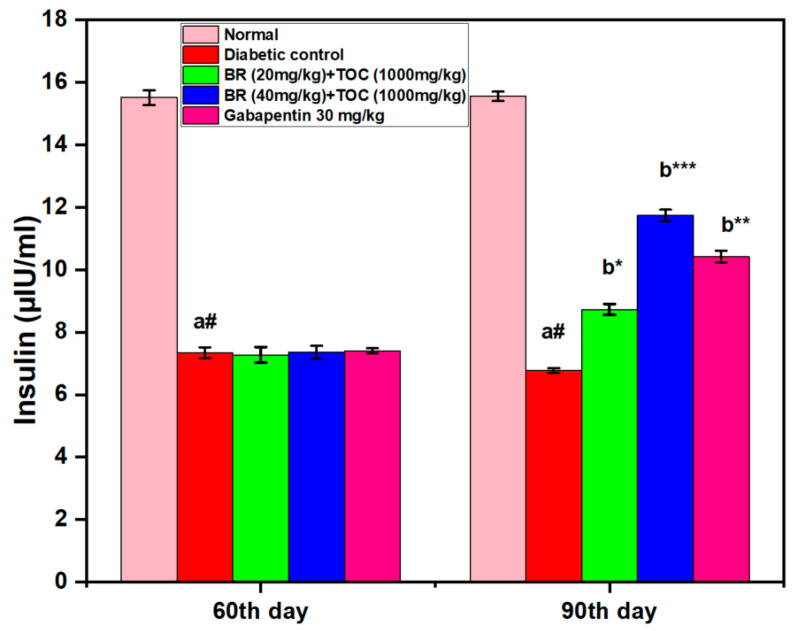
Effect of Berberine and Tocopherol on serum insulin level. Values are represented as mean ± SEM. (a#): normal control vs. diabetic control. (b*): diabetic control vs. 20 mg/kg and 40 mg/kg of BR (Berberine) with 1000 mg/kg of TOC (Tocopherol) and 30 mg/kg of the standard drug (Gabapentin). (***): *p* < 0.001, (**): *p* < 0.01, and (*): *p* < 0.05.

This study finds a significant decrease in serum insulin levels among diabetic control compared with normal control after 90 days (6.773 ± 0.07 μIU/mL vs. 15.55 ± 0.15 μIU/mL, *p* < 0.001). However, when administered with BR + TOC at doses of 20 mg/kg and 40 mg/kg and 1000 mg/kg for the same period, there is a notable increase in serum insulin level, which measures up to 11.73 ± 0.18 μIU/mL (*p* < 0.001) (Figure 4).

### 3.4. Effect on Thermal Hyperalgesia

**Figure 5:**
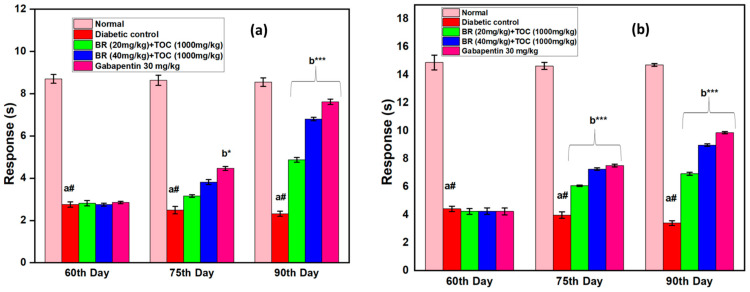
(**a**,**b**) Effect of Berberine and Tocopherol on thermal hyperalgesia. Values are represented as mean ± SEM. (a#): normal control vs. diabetic control. (b*): diabetic control vs. 20 mg/kg and 40 mg/kg of BR (Berberine) with 1000 mg/kg of TOC (Tocopherol) and 30 mg/kg of the standard drug (Gabapentin). (***): *p* < 0.001 and (*): *p* < 0.05.

In DN rats, there is a statistically significant drop in the nociceptive pain threshold when tested using the hot-plate or the tail immersion method (Figure 5a,b). After 60 days of STZ treatment using either the hot plate or the tail immersion approach, hyperalgesia was observed in the animals. Compared with DN control rats, administration of BR + TOC (20 mg/kg and 40 mg/kg + 1000 mg/kg) generate a dose- and time-dependent increase in pain threshold. Furthermore, compared with the diabetic control group, diabetic rats given Gabapentin at a dose of 30 mg/kg dramatically increased their threshold for withdrawal of the paw.

### 3.5. Effect on Mechanical Hyperalgesia

**Figure 6:**
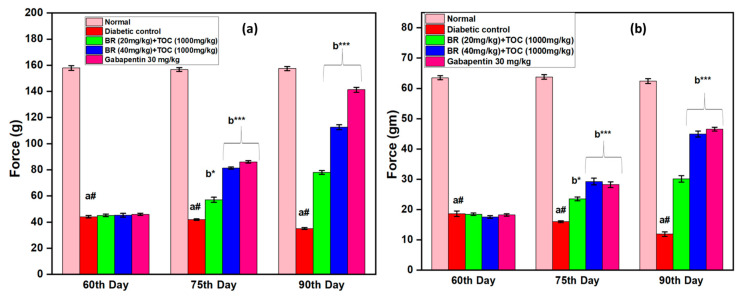
(**a**,**b**) Effect of Berberine and Tocopherol on mechanical hyperalgesia. Values are represented as mean ± SEM. (a#): normal control vs. diabetic control. (b*): diabetic control vs. 20 mg/kg and 40 mg/kg of BR (Berberine) with 1000 mg/kg of TOC (Tocopherol) and 30 mg/kg of the standard drug (Gabapentin). (***): *p* < 0.001 and (*): *p* < 0.05.

At the end of the 90th day of the trial, the Randall–Sellito threshold for paw withdrawal and the von Frey threshold for tactile withdrawal (Figure 6a,b) due to light touch are significantly lower compared with the DN control. In both experiments, treatment with BR + TOC (20 mg/kg and 40 mg/kg + 1000 mg/kg) reverses the decrease in the threshold for withdrawal of the paw that diabetes caused compared with the DN control group. Compared with the groups treated with BR + TOC (20 mg/kg and 40 mg/kg + 1000 mg/kg) treated groups, the attenuation of the lower mean withdrawal threshold of the paw caused by Gabapentin (30 mg/kg, i.p.) is much more substantial (*p* > 0.001) when administered by Gabapentin.

### 3.6. Effect on Antioxidant Enzymes (GSH and SOD)

**Figure 7:**
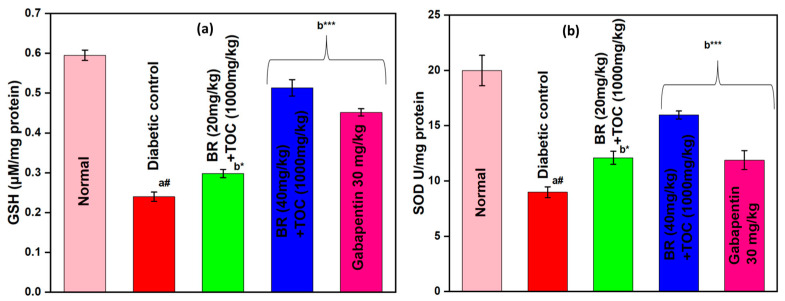
(**a**,**b**) Effect of Berberine and Tocopherol on antioxidant enzymes; (**a**) GSH and (**b**) SOD. Values are represented as mean ± SEM. (a#): normal control vs. diabetic control. (b*): diabetic control vs. 20 mg/kg and 40 mg/kg of BR (Berberine) with 1000 mg/kg of TOC (Tocopherol) and 30 mg/kg of the standard drug (Gabapentin). (***): *p* < 0.001 and (*): *p* < 0.05.

BR + TOC (20 mg/kg and 40 mg/kg + 1000 mg/kg) increases GSH to 0.30 ± 0.01 and 0.51 ± 0.02 μM/mg protein compared with diabetic control (0.24 ± 0.01 μM/mg protein) (Figure 7a).

BR + TOC (20 mg/kg and 40 mg/kg + 1000 mg/kg) improves the level of SOD to 12.09 ± 0.24 and 15.96 ± 0.15 U/mg protein when related to diabetic control group (8.98 ± 0.19 U/mg protein) (Figure 7b).

### 3.7. Effect on Lipid Peroxidation

**Figure 8:**
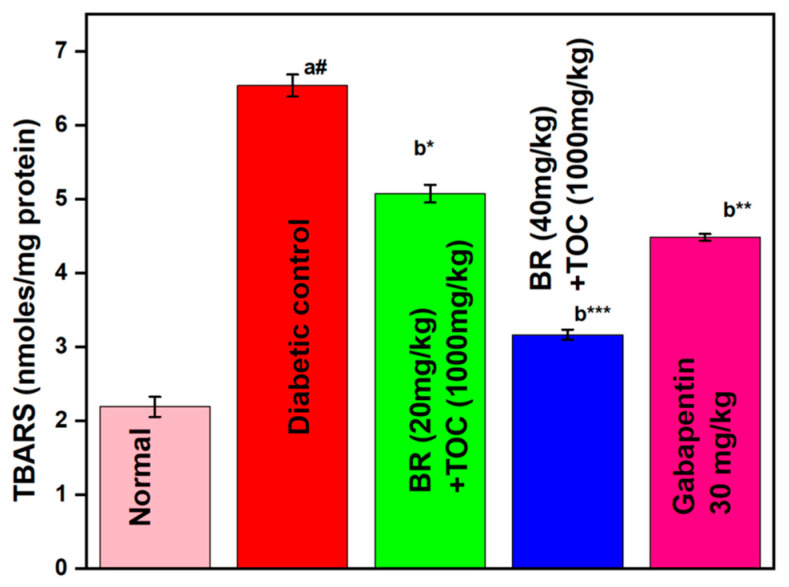
Effect of Berberine and Tocopherol on lipid peroxidation. Values are represented as mean ± SEM. (a#): normal control vs. diabetic control. (b*): diabetic control vs. 20 mg/kg and 40 mg/kg of BR (Berberine) with 1000 mg/kg of TOC (Tocopherol) and 30 mg/kg of the standard drug (Gabapentin). (***): *p* < 0.001, (**): *p* < 0.01, and (*): *p* < 0.05.

BR + TOC (20 mg/kg and 40 mg/kg + 1000 mg/kg) decreases the TBARS level to 5.07 ± 0.12 and 3.16 ± 0.069 nmol/mg protein when related to the diabetic control (6.53 ± 0.15 nmol/mg protein) (Figure 8).

### 3.8. Effect on AGEs

**Figure 9:**
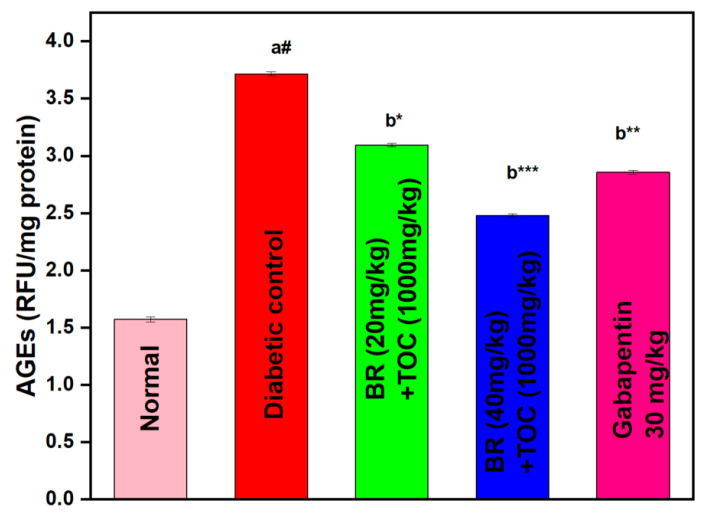
Effect of Berberine and Tocopherol on AGEs. Values are represented as mean ± SEM. (a#): normal control vs. diabetic control. (b*): diabetic control vs. 20 mg/kg and 40 mg/kg of BR (Berberine) with 1000 mg/kg of TOC (Tocopherol) and 30 mg/kg of the standard drug (Gabapentin). (***): *p* < 0.001, (**): *p* < 0.01, and (*): *p* < 0.05.

Due to diabetes, there is a substantial increase in the level of AGE in the kidney in comparison with normal rats. Administration of BR + TOC (20 mg/kg and 40 mg/kg + 1000 mg/kg) significantly (*p* < 0.001) alleviates AGE levels in the kidney when related to diabetic control rats (3.72 ± 0.02 RFU/mg protein). The doses of BR + TOC (50 mg/kg and 100 mg/kg + 1000 mg/kg) doses reduce the AGEs to 3.09 ± 0.02 and 2.48 ± 0.02 RFU/mg protein (Figure 9).

### 3.9. Effect on Nitrite Level

**Figure 10:**
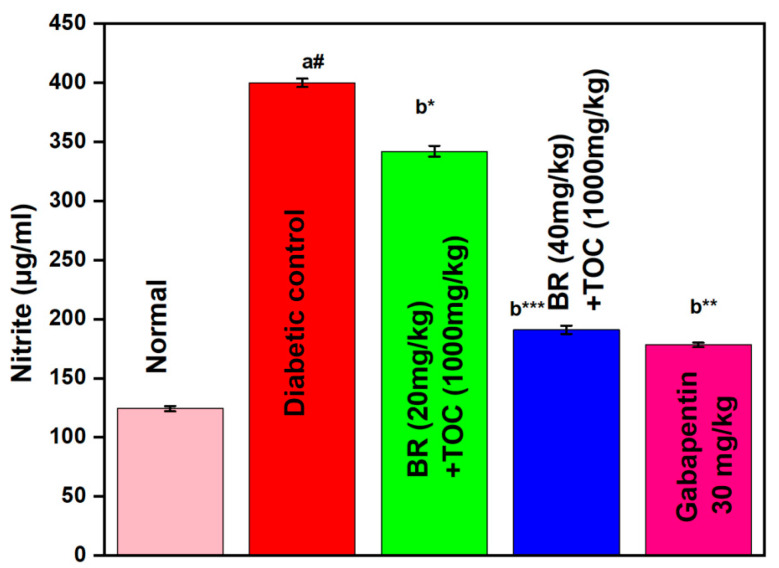
Effect of Berberine on nitrite level. Values are represented as mean ± SEM. (a#): normal control vs. diabetic control. (b*): diabetic control vs. 20 mg/kg and 40 mg/kg of BR (Berberine) with 1000 mg/kg of TOC (Tocopherol) and 30 mg/kg of the standard drug (Gabapentin). (***): *p* < 0.001, (**): *p* < 0.01, and (*): *p* < 0.05.

The diabetic rat showed a remarkable increase in the amount of nitrite detected within the sciatic nerve. However, the administration of Berberine and Tocopherol at doses of 20 mg/kg + 1000 mg/kg and 40 mg/kg + 1000 mg/kg for 90 days results in significant prevention of this elevation in nitric oxide levels (*p* < 0.001) when compared with the DN control group. Furthermore, Gabapentin proves to be even more effective than Berberine and Tocopherol in preventing the treated groups with lower amounts of nitrite at the two doses mentioned above (Figure 10).

### 3.10. Effect on NF-kB and TNF-α

**Figure 11:**
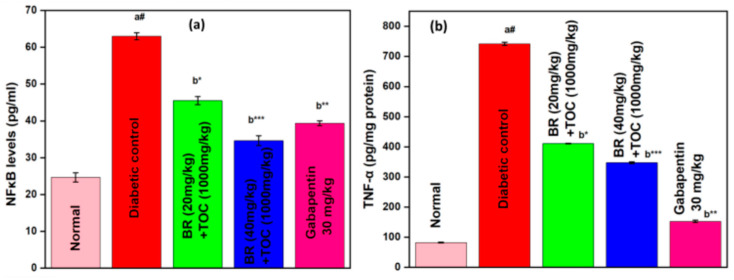
(**a**,**b**) Effect on NF-kB and TNF-α. Values are represented as mean ± SEM. (a#): normal control vs. diabetic control. (b*): diabetic control vs. 20 mg/kg and 40 mg/kg of BR (Berberine) with 1000 mg/kg of TOC (Tocopherol) and 30 mg/kg of the standard drug (Gabapentin). (***): *p* < 0.001, (**): *p* < 0.01, and (*): *p* < 0.05.

The combination of Berberine and Tocopherol has a persuasive effect in reducing the levels of inflammatory mediators (as shown in Figure 11). In rats with streptozotocin-induced diabetes, there was an increase in levels of the NF-kB protein (Figure 11a) and TNF-α (Figure 11b). The diabetic control group shows a significant increase in renal NF-kB level (63.0 ± 0.973) and TNF-α level (741.95 ± 5.44) when compared with normal subjects. However, treatment with Berberine and Tocopherol at doses of 20 mg/kg + 1000 mg/kg or 40 mg/kg + 1000 mg/kg for three months significantly reduces raised levels of both NF-kB (*p* < 0.001) and TNF-α (*p* < 0.001), demonstrating their powerful ability to effectively inhibit inflammation against diabetic control groups acting as references.

## 4. Discussion

Diabetes-induced neuropathy is a frequently occurring ailment that affects the peripheral nervous system, often accompanied by distressing sensations such as discomfort, lack of sensation, and reduced sensory function [69]. Emerging evidence suggests that gut microbiota alters its development [70]. Altered intestinal microbiota diversity is observed in diabetic neuropathy, characterised by reduced beneficial bacteria and an overgrowth of pathogenic strains. This dysbiosis may exacerbate symptoms through inflammation and metabolic dysfunction [10]. Changes in the gut microbiota can cause inflammation, leading to diabetic neuropathy. Inflammatory molecules directly damage the peripheral nerves [71].

Our research reveals that the combination of Berberine and Tocopherol effectively reduces glucose levels in fasting rats by targeting both the antihyperglycaemic and NF-κB regulatory pathways. This is particularly important as hyperglycaemia triggers the formation of AGEs, which lead to cell death when bound with receptors and producing ROS. Additionally, activation of nuclear factor kappa B through receptor ligation causes inflammation-related gene expression, further exacerbating neurodegenerative diseases. Given its central role in immune and inflammatory responses, mounting evidence suggests that the transcription factor NF-kB contributes to the onset and progression of the disease [72,73]. Various factors such as oxidative stress, bacterial and viral infections, growth factors, and cytokines can induce activation of the NF-kB transcription factor in the brain. Subsequently, it enters the nucleus and latches onto specific DNA segments that lead to gene transcription that promotes inflammation, including chemokines, cytokines, and adhesion molecules, amongst other inflammatory genes. Animal models with neuropathy have shown that mitigating NF-kB activation results in less severe damage to their brains; thus, confirming that targeting this signalling could be a useful therapeutic approach for various conditions that affect our cognitive system [74].

Current research has shown that elevated levels of NF-κB and TNF-α can be reduced by administering various doses of BR + TOC (20 mg/kg and 40 mg/kg + 1000 mg/kg). This demonstrates the potential of these substances to alleviate inflammatory mediators. The reduction in dyslipidaemia may possibly be attributed to its antihyperglycaemic and anti-inflammatory properties, along with an increase in insulin production from pancreatic β-cells. Furthermore, diabetic rats exhibited a significantly lower nociceptive threshold, as evidenced by hot-plate, tail immersion, Randall–Selitto and von Frey hair tests. The results suggest that not only do diabetic animals experience tactile allodynia, but they also exhibit heightened sensitivity to heat and mechanical stimuli. To address this issue, the administration of Berberine, along with Tocopherol in varying doses, proved to be an effective solution for managing symptoms of thermal and mechanical hyperalgesia.

In diabetic neuropathy, diabetic rats showed characteristic weight loss due to the insufficient level of insulin that prevented glucose transportation to cells. Hence, the body starts to use fats and muscles for energy leading to loss of body weight. There is a significant decrease in glucose level and an improvement in body weight after Berberine administration in combination with Tocopherol, which may be elucidated by activation of pancreatic β-cells that have been left over or their regrowth after STZ has partially killed them. The increased level of insulin secretion and rise in peripheral glucose use may also contribute to the antihyperglycaemic action of Berberine in combination with Tocopherol. The administration of Berberine and Tocopherol decreased the frequency of weight loss in rats with diabetic neuropathy at the end of the study. Excess glucose enters the fatty acid production pathways, and glycogenesis is inhibited in insulin insufficiency.

Superoxide dismutase (SOD) and glutathione (GSH) are two of the most well-known antioxidant enzymes that help protect cells from free radical damage, and GSH and SOD activity was found to be reduced by hyperglycaemia in diabetic rats. It is also hypothesised that superoxide anions produce an increase in protein kinase C and aldose reductase activity, both of which are further implicated in the experience of pain. When superoxide anions are converted into hydrogen peroxide, SOD offers antioxidant protection to cells. Diabetes is associated with changes in GSH metabolism, which may possibly contribute to an increase in TNF-α and IL-1b. The levels of superoxide dismutase and glutathione peroxidase in the sciatic nerve of diabetic mice were dramatically reduced; however, these levels were recovered by treatment with Berberine and Tocopherol. Furthermore, the total amount of nitrous oxide, often known as NO, which is an indication of nitrosative stress, is elevated in the experimental model of DN. Peroxynitrite is created when NO and superoxide combine to quickly induce DNA damage, cell death, and protein nitration or nitrosylation.

Furthermore, the damaging effects of high glucose levels on nerve tissue are a significant cause of neuropathic pain in diabetic patients. Overproduction of nitric oxide (NO) compounds this issue by exacerbating oxidative stress and further contributing to nerve damage. However, promising results have been observed through treatment with Berberine and Tocopherol at varying dosages, leading to reductions in elevated NO levels within the blood. The underlying culprits behind these complications lie primarily within hyperglycaemia-induced oxidative stress, a state that leads to excessive free radical production resulting in severe consequences for DNA, RNA, lipids, and proteins. These detrimental effects can manifest themselves through various pathological conditions, including diabetic neuropathy. Fortunately, recent studies demonstrate that Berberine and Tocopherol hold immense potential as neuroprotective agents against such harmful outcomes. Through inhibition of NF-κB signalling pathways, they exhibit potent activity toward preventing or reversing diabetic complications associated with neural dysfunction.

## 5. Conclusions

Through an exemplary in vivo investigation, diligent researchers have brought to light captivating revelations about the intrinsic capabilities of this molecule. The remarkable findings not only confirm its efficacy but also reveal that it has extraordinary potential to alleviate some of the most pressing factors associated with diabetes, including hyperglycaemia, oxidative stress, and inflammatory pathways such as NF-κB. These fundamental elements frequently aggravate diabetic complications, elevating this discovery above all others as a groundbreaking development with immense implications for people struggling with this incapacitating ailment.

The findings of this study reveal a fascinating connection between Berberine and Tocopherol, demonstrating their ability to significantly impede the advancement of diabetic neuropathy. By skilfully controlling hyperglycaemia levels while simultaneously suppressing inflammation induced by numerous agents such as NF-κB, these two compounds seem to have immense potential to aid in holistic management for diabetes patients. The implications are vast and encourage further research on how these components can become indispensable targets when dealing with diabetes treatment in a comprehensive way.

## Data Availability

The data presented in this study are available upon request from the corresponding author.
